# Iranian Pediatric COVID-19 Epidemiology and Clinical Characteristics

**DOI:** 10.1155/2021/4914371

**Published:** 2021-12-15

**Authors:** Shahnaz Armin, Mohammadreza Mirkarimi, Zahra Pourmoghaddas, Marjan Tariverdi, Azadeh Jafrasteh, Noushin Marhamati, Armin Shirvani, Abdollah Karimi, Sedigheh Rafiei Tabatabaei, Roxana Mansour Ghanaei, SeyedAlireza Fahimzad, Fariba Shirvani, Seyedeh Mahsan Hoseini-Alfatemi

**Affiliations:** ^1^Pediatric Infections Research Center, Research Institute for Children's Health, Shahid Beheshti University of Medical Sciences, Tehran, Iran; ^2^Aboozar Children's Medical Center, Ahvaz Jundishapur University of Medical Sciences, Ahvaz, Iran; ^3^Pediatrics Infectious Disease Department, Isfahan University of Medical Sciences, Isfahan, Iran; ^4^Department of Pediatric, Clinical Research Development Center of Children Hospital, Hormozgan University of Medical Sciences, Bandar Abbas, Iran; ^5^Department of Pediatrics, School of Medicine, Lorestan University of Medical Sciences, Khorramabad, Iran; ^6^Virtual School of Medical Education and Management, Shahid Beheshti University of Medical Sciences, Tehran, Iran

## Abstract

**Background:**

Despite the worldwide spread of Severe Acute Respiratory Syndrome Corona Virus 2 (SARS-CoV-2), information about the epidemiological and clinical patterns of this infection is still largely unknown in children. In addition, the prevalence of this disease is still very high in some parts of the world, including Iran. Thus, this study aims to evaluate the epidemiological features, laboratory and imaging findings, and the type of treatments in children with novel coronavirus 2019 (COVID-19).

**Method:**

This study is conducted from March 2020–March 2021 by using the medical records of hospitalized confirmed COVID-19 children younger than 18 years in five cities of Iran: Tehran, Ahwaz, Isfahan, Bandar-Abbas, and Khorramabad. In addition to demographic and epidemiological data, we also studied clinical signs and treatments.

**Results:**

In total 278 confirmed COVID-19 children, the average age was 5.3 years, and 59.4%were boys. A total of 37.8% had an underlying disease, in which the most common was a malignancy. The most common symptoms were fever and cough. In this group of pediatrics, some abnormal laboratory findings have been seen. GGO (Ground-Glass Opacity) had been diagnosed in 58.6% of children. 3.6% needed oxygen therapy with ventilators, and 83.09% had received antibiotic treatments with the majority of ceftriaxone. Also, 10% had got steroids. In this study, the mortality rate was 4.3%.

**Conclusion:**

In this study, most of the children who died had an underlying disease, so timely care and action is important in them. Most children admitted to our study received antibiotics and were prescribed antivirals and steroids for a smaller number. Also, a small number of children received oxygen therapy, most of whom were in the age group of 1 to 5 years.

## 1. Introduction

Coronavirus disease 2019 (COVID-19) is caused by severe acute respiratory syndrome coronavirus-2 (SARS-CoV-2). Because of the intensity and alarming level of spread, the World Health Organization (WHO) deemed it a pandemic [1]. The first pandemic infection to occur due to a coronavirus [2], SARS-CoV-2, is a factor that effects all age groups.

COVID-19 has a low incidence of severe cases among children. Children have a milder clinical course than adults [3–6]. Children and adults have different levels of immune maturity, which may be the reason for differences in the prevalence and type of clinical manifestations of COVID-19 [7]. However, it is also important to note that even mild COVID-19 can cause transmission [8]. According to the work of Gandhi et al., asymptomatic transmission is the Achilles' heel of this pandemic [9].

In other words, children can cause cluster propagation in the home environment [10]. Furthermore, children with gastrointestinal symptoms can transmit the virus through their feces for weeks, which is dangerous in some places such as kindergartens or elementary schools [11–13].

In addition to acute COVID-19 in children, there are several reports of multisystemic involvement, which complicates the diagnosis and management of this disease in pediatrics.

First cases of COVID-19 were observed in Iran in January 2020, but the country began reporting cases to the WHO on February 2020 [14]. Since then, the disease has spread rapidly across the country, infecting many children [5]. The purpose of this study was to determine the clinical presentation and treatment pattern of COVID-19 among children admitted to 5 major cities in Iran.

## 2. Method

We conducted a retrospective, cross-sectional, multicenter study from March 2020 to March 2021 in several Iranian cities (Tehran, Ahwaz, Isfahan, Bandar-Abbas, and Khorramabad). During the study period, all children hospitalized with confirmed SARS-CoV-2 infection in one of the participating centers were included. Data were extracted from medical records of children younger than 18 years diagnosed with confirmed cases of COVID-19 with positive real-time reverse transcription polymerase chain reaction (RT-PCR) results on nasopharyngeal samples. Besides demographic and epidemiological data, we also studied clinical symptoms and treatments. Data were collected from medical forms and records by a pediatrician. No written consent has been obtained from the patients as there are no patient identifiable data included in this research.

The research ethics committee of Shahid Beheshti University of Medical Sciences approved the study (Ethical code: IR.SBMU.RICH.REC.1400.007).

## 3. Result

**Figure 1:**
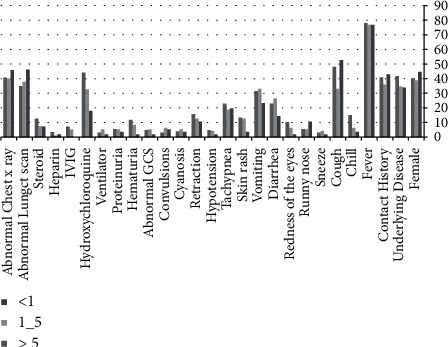
General information and clinical symptoms of pediatric patients with confirmed COVID-19 in 3 age groups (<1, 1–5, >5).

The average age of the 278 children and adolescents hospitalized with COVID-19 was 5.3 years. Of the 278 patients, 56 (20.1%) were under one year of age, 95 (34.2%) were between 1 and 5 years of age, and 127 (45.7%) were over five years of age. There were 59.4% boys among the cases. Results of the study, which include general information and clinical signs, are divided into 3 age groups (under 1 year, 1 to 5 years, and over 5 years) in Figure 1.

A total of 37.8% of children hospitalized had an underlying disease. By far, the most common condition was malignancy (10%), followed by diabetes (5%). We estimate that the mortality rate was 4.3% (12 patients) as 8 of them had an underlying disease, and half of the dead were over 5 years of age. Approximately 40% of our study population was exposed to an indicator case or an adult with suspected SARS-CoV-2 infection. 3.6% of hospitalized children needed oxygen therapy with ventilators, most of whom were 1 to 5 years old.

Children with COVID-19 tend to experience fewer symptoms than adults. Fever was the most common symptom (77.3%), according to the information obtained. Cough was another symptom (43.8%). Both diarrhea and vomiting were present in 22.3% and 30.3% of cases, respectively. In 20.9% of cases, tachypnea was reported. Infection was also associated with sneezing and runny nose in 3.2% and 6.5% of children, respectively. A skin rash was reported in 11.2% of children hospitalized with COVID-19 and red eyes in 7.2%. The mean O2 level was 93.6% (with a minimum of 68 and a maximum of 99%). O_2_ was below 93% in 22.6% of cases. Frequently, children hospitalized for illnesses have elevated inflammatory markers, such as ESR, CRP, and liver enzyme. The mean initial CRP in the under one age group was lower than in other age groups. BUN and Cr were abnormal in 13.6% and 8% patients, respectively. In our research, based on the division of LDH into two groups above 500 and above 1000, patients were included in 56.88 and 8.3%, respectively. Abnormal AST and ALT were found in 37.17% and 15.03%, respectively. Laboratory findings are presented in Table 1. In our review, leukocytosis had been reported in 24.5% of the patients, while 9.33% of our pediatrics had leukopenia. Different age groups have reported lymphopenia: 44% under one year of age, 28.04% in 1 to 5 years, and 34.25% in people 5 years and older. GGO has been diagnosed in 58.6% of children with lung involvement, mainly in RLL and LLL.

A total of 231 patients (83.09%) were treated with antibiotics, with the most common drug used being ceftriaxone (161), followed by vancomycin (67), meropenem (48), and azithromycin (42), given as a two- or three-medicine regimen. There were also 100 patients given antivirals (35.97%), most of whom were given Kaletra (52 patients) and atazanavir (22 patients). Also 10% had steroid treatments.

## 4. Discussion

In Wenjun et al.'s retrospective study, the median age was 6.2 years [15], while in ours, the mean age was 5.3 years. The study showed that boys made up the majority (59.4%), as in other studies including the work of Dong et al. (56.6%) [5]. Hua et al. in an epidemiological study reported the mean age in children was 8.16 years (in the range of 3.66 months–14 years) and 60.5% were male [16].

In the study by Tezer and Bedir Demirdağ, among 345 confirmed children, 23% had underlying diseases and the most common ones were chronic pulmonary disease (counting asthma), cardiovascular disease, and immunosuppression (caused by cancer, chemotherapy, etc.) [17]. In addition, in Kompaniyets et al.'s cross-sectional study, among 43,465 patients with COVID-19 pediatrics, 28.7% had underlying medical conditions, mostly asthma, neurodevelopmental disorders, anxiety-related disorders, depressive disorders, and obesity [18]. Also, we found that 37.8% of our hospitalized children had an underlying disease with the most common being malignancy followed by a chronic disease such as diabetes. The differences likely arise from the type of underlying conditions that researchers are searching for.

Hoang et al.'s study had reported 0·09% deaths among the COVID-19 children [19], while by our results, the estimated mortality rate was below 5 percent.

According to Chang et al.'s systematic literature (with last updates on 15 March 2020), as well as Ansel et al.'s systematic search (with last searched 14 May 2020) and Hoseinyazdi et al.'s pediatric study in Shiraz (March–May 2020), like ours, fever and cough were the most common clinical symptoms [19–21]. Although Chang et al.'s study reported few gastrointestinal symptoms (12%) [20], in our study, diarrhea and vomiting were reported in 22.3% and 30.3% of cases, respectively. Possibly because we studied at a different time and place, the predominant virus strain may have been different.

Two systematic reviews have shown that most children with COVID-19 have a normal WBC count and the most common abnormality is leukopenia [22, 23]; this *variance* may be due to differences in age group or severity of the infection or virus.

In our review, 24.5% of the patients had leukocytosis, while only 9.33% had shown leukopenia. In a systematic review by Henry et al., leukocyte counts were normal in most children, and lymphopenia was present in only 3%, none of whom had severe disease [24].

In our investigations, out of 240 patients, 34.16% had been shown with lymphopenia and only 2% had lymphocytosis. Based on Hoseinyazdi et al.'s pediatric study in Namazi and Ali-Asghar Hospitals, lymphocytosis has been shown in severe cases [21]. Additionally, another study by Du et al. in Shandong Province in China found increased lymphocyte counts in children compared to adults [15]. According to Kosmeri et al., lymphocytosis was the most common findings in neonates and infants with COVID-19. Moreover, anemia and thrombocytopenia were rarely seen in COVID-19 children [25].

In Hoseinyazdi's study, CRP levels were normal in patients under two years of age, whereas they were significantly higher in those aged over 3 years [21]. Also, in our study, the mean range of initial CRP was lower in the group under 1 years of age than that of those over this age.

Abnormal levels of LDH were seen in fifty percent of children with COVID-19 in the study by Du et al. and also showed that positive LDH levels were significantly higher in children than in adults [15]. In ours, more than half of the study population had an LDH above 500 and less than a tenth had it above 1000. Furthermore 37.17% and 15.03% had abnormal AST and ALT in that order; while in Esmaeili et al.'s retrospective study, 27.8% and 38.9% of cases showed abnormally high ALT and AST levels, respectively [26].

According to an *Italian* report from an emergency department for children, 4% of children had oxygen saturation below 95%. All of these patients also had imaging evidence of lung involvement [4]. In our observations, the mean O_2_saturation level was 93.66% and O_2_sat was below 93% in 22.6% of reported cases. Since our data pertain to sick patients admitted to hospitals, the figure is higher than Italian rates, even though most Italian patients had not been in a bad situation.

The most common radiographic finding in Chang et al.'s investigations was ground-glass opacities (48%) [20]. In Samy and Khalaf's investigations, 44 (83%) patients had normal CT and only 9 patients presented lung opacities in which 5 cases showed consolidation and 2 cases were with GGO, while in another 2 cases, consolidation with GGO was noted. The most involved lobes were the right and left lower lobes [27]. In our study, GGO was the most commonly reported finding in 58.6% of children with pulmonary involvement (in RLL and LLL).

In one of the observational studies in Wuhan, China, six of the eight patients received high-flow oxygen therapy and two critically ill patients were mechanically ventilated. Antiviral therapies (Verazole, oseltamivir, and interferon) were administered to all patients. Antibiotics (in 5/8), traditional Chinese medicine (in 4/8), intravenous immunoglobulins (in 4/8), and glucocorticoids (in 5/8) were also used according to the children's condition [28].

In our education, a total of 231 patients (83.09%) received antibiotic treatment, the majority of which were ceftriaxone. vancomycin, meropenem, and azithromycin; on the other hand, they were also commonly administered in combination with each other. A total of 100 patients were also prescribed antiviral medicines, the majority of whom took Kaletra or atazanavir based on national protocols at the time of the study. In addition, about 10% of our patients received steroid medication. Fewer than 4% of hospitalized children required respiratory oxygen therapy, most of whom were between the ages of 1 and 5 years.

Based on a retrospective study by Zhang et al., ribavirin was given to 44% of patients. 85% had received antibiotic therapy. Corticosteroids (15%) and supportive oxygen inhalation therapy (9%) were also used [29].

## 5. Conclusions

Among the children who died in this study, the majority had the comorbidities. In order to protect children with underlying diseases, care and isolation must be provided in a timely manner. Cough and fever were the most common clinical symptoms. Leukocyte changes, especially lymphopenia, have been reported less frequently in children under 1 year of age with COVID-19, possibly due to their immature immune systems and ACE2 expression. Most patients received antibiotics, and relatively fewer antivirals and steroids were administered. Also, oxygen therapy was used to a much lesser extent in our patients, most of whom were in the age group of 1–5 years. Therefore, paying attention to the abovementioned results can help us reduce the prevalence of this disease.

## Figures and Tables

**Table 1:** Laboratory findings of the hospitalized COVID-19 pediatric patients.

Laboratory data	Number	Minimum	Maximum	Mean	Std. deviation

O_2_sat in room temperature	137	68	99	93.66	5.379
WBC	257	120	43600	8979.96	5415.383
ALC	242	86	16947	3063.30	2527.806
Hb	257	4.50	17.70	11.12	2.068
PLT	256	6000	838000	271807.46	144975.231
CRP	208	0	178	19.28	27.452
ESR	226	1	210.7	26.90	27.78
BS	201	1	891.5	124.93	96.95
BUN	249	3	1542	24.11	110.221
Cr	249	0.10	54	1.04	3.584
SGOT	155	5	232	42.62	31.750
SGPT	152	3	1478	36.53	119.919
ALK.P	103	93	1822	477.08	299.025
PT	78	10	32	13.70	4.114
PTT	74	12.20	70	32.74	9.504
INR	77	0.78	1.50	1.07	0.139
LDH	167	6.90	2697	623.62	387.434
Ferritin	65	4	1714	311.25	386.216
Fibrinogen	20	67	1000	330.30	196.052
CPK	113	16	2126	155.53	230.546
D.dimer	45	0.09	3327	581.64	776.268
Troponin	53	0	28.80	5.03	6.903
PH	81	6.91	7.70	7.34	0.143
PCO2	81	14	98	35.53	12.199
HCO3	80	5.70	27.70	19.35	4.323

## Data Availability

The authors declare the data used to support the findings of this study are available from the corresponding author upon request.

## Additional Points

Because this is a retrospective study that collected information from medical records in different cities, some data and variables were not available; in addition, it was not possible to complete it (for example, some tests were not performed in all patients).
